# Bovine Tuberculosis Management in Northwest Minnesota and Implications of the Risk Information Seeking and Processing (RISP) Model for Wildlife Disease Management

**DOI:** 10.3389/fvets.2018.00190

**Published:** 2018-08-17

**Authors:** Megan Cross, Alex Heeren, Louis J. Cornicelli, David C. Fulton

**Affiliations:** ^1^Department of Fisheries and Wildlife, Michigan State University, East Lansing, MI, United States; ^2^Sierra Nevada Research Institute, University of California, Merced, CA, United States; ^3^Minnesota Department of Natural Resources, Division of Fish and Wildlife, St. Paul, MN, United States; ^4^U.S. Geological Survey, Minnesota Cooperative Fish and Wildlife Research Unit, University of Minnesota, St. Paul, MN, United States

**Keywords:** bovine tuberculosis, communication, disease management, information-seeking, risk information seeking and processing (RISP) model

## Abstract

Bovine tuberculosis (bTB) is an infectious, zoonotic disease caused by *Mycobacterium bovis* that can spread between domestic and wild animals, as well as to humans. The disease is characterized by the progressive development of lesions that compromise the victim's lungs and lymph system. The disease was first identified in northwest Minnesota in both cattle and white-tailed deer (*Odocoileus virginianus*) in 2005. Due to its risks to human and animal health, bTB has numerous implications related to population management, policy outcomes, stakeholder relations, and economic impacts. When dealing with complicated risks, like bTB, individuals often seek out and process information as a method to learn about, and cope, with the risk. We developed a questionnaire that adapted components of the Risk Information Seeking and Processing (RISP) model and surveyed northwest Minnesota deer hunters. Our objectives were to better understand how stakeholders perceive and act on information regarding disease management in wildlife and to understand the utility of the RISP model for such management contexts. We drew a random proportional sample of licensed deer hunters (*n* = 2100) from the area affected by bTB and conducted a multi-contact mail survey. We found that 43% of the variability in the information-seeking behaviors of respondents was explained by demographics, hunting importance, personal risk perceptions, attitudes, and subjective norms. However, these results are largely attributable to the factors in the RISP model encompassed by components of the Theory of Planned Behavior (i.e., attitudes, subjective norms, perceived behavioral control, and behavioral intentions). This information can help managers contextualize individuals' perceived risks to better frame communication efforts to address stakeholder concerns and develop best practices for disease communication. While the state of Minnesota is currently considered free of bTB, future outbreaks remain possible in Minnesota and elsewhere. Understanding the key factors in the processes through which deer hunters seek out information pertaining to the disease can help managers collect the data necessary to aid decisions about desired future management outcomes. In addition, testing RISP model performance in applied research improves its future use across a broad spectrum of topics throughout veterinary disease management.

## Introduction

Bovine tuberculosis (*Mycobacterium bovis*, hereafter referred to as “bTB”) is an infectious zoonotic disease that can spread among domestic and wild mammals and, in rare cases humans. Zoonotic diseases like bTB threaten agricultural economies, pose health risks to humans and wildlife, and disturb the social, political, and economic environments where they occur. In Minnesota, the appearance of bTB among wild white-tailed deer (*Odocoileus virginianus*) in 2005 was not only a health concern but also posed a risk to the state's deer hunt, a major economic industry, cultural event, and the primary method in which the state manages its deer population. Therefore, as bTB engaged numerous stakeholder groups, understanding how hunters viewed the risk, and whether their concerns impacted the decision to hunt, was of particular interest to management agencies.

The human dimensions of disease management in wildlife has increased in importance during recent years (1–5). Following Clarke (1), we used the Risk Information Seeking and Processing model (RISP) (6) as a core framework to discern the key considerations for understanding and better communicating with stakeholders about disease management in wildlife. In this study, we were interested in delineating the processes through which Minnesota deer hunters sought information about risks from bTB.

We addressed the following key questions:

1. How do hunters seek information about bTB?
2. What factors affect hunters' information seeking behaviors?
3. Is the RISP model useful for understanding well-established wildlife disease management issues?
4. What are the implications for natural resource management agencies and professionals?

Such information can help guide managers' decisions regarding the collection and analysis of information related to individuals' perceived risks and improve the development of communication best practices in instances of disease in wildlife.

The initial detection of bTB in northwest Minnesota occurred at a beef cattle operation in 2005 (7). Upon further testing, the disease was found in several other beef cattle operations and detected in wild white-tailed deer (*Odocoileus virginianus*) during fall 2005 hunter-harvested surveillance (7). Epidemiological evidence indicated the disease was introduced into a single beef cattle operation and from there it spilled over to deer (8, 9). Deer presumably served as a spillover host for the transmission of the disease among area livestock operations (8, 10, 11).

In response to the detection of bTB in cattle and deer, the Minnesota Department of Natural Resources (DNR) and the Minnesota Board of Animal Health took joint actions to decrease the likelihood of disease spread, eradicate the disease from cattle (and thus regain Accredited-free status within the United States Department of Agriculture's bTB eradication program), and reduce wild deer prevalence to undetectable levels. These strategies centered on preventative measures to reduce the likelihood of disease transmission. Example strategies included a temporary buy-out of cattle producers, construction of deer exclusion fencing around stored forage, prohibiting recreational deer feeding, and reducing the local deer population using hunting regulations, aerial gunning, ground sharpshooting, and deer shooting permits issued to landowners (8, 12, 10).

Although the disease was successfully eradicated in cattle and reduced to an undetectable level in deer by 2012, deer hunters considered the actions taken to achieve bTB-free status controversial (12). Among the general public, lethal control of deer (sharpshooting) is often contentious (12–14). Further, in instances of zoonotic disease affecting game species, hunters show more concern than the general public about game management (15, 16).

As it was being used in other studies the RISP model is the primary source for the conceptual framework used in our study, and data were collected following the methods outlined in Griffin et al. (6) work on RISP and recent adaptations to that model [see 1)]. The RISP model builds upon an earlier model, the Theory of Planned Behavior, by examining the relationship between information, knowledge, and risk perception (6). Multiple studies in the field of risk and threat perception have shown that information seeking and processing is an important component of how an individual perceives and responds to a risk (17–19).

**Figure 1:**
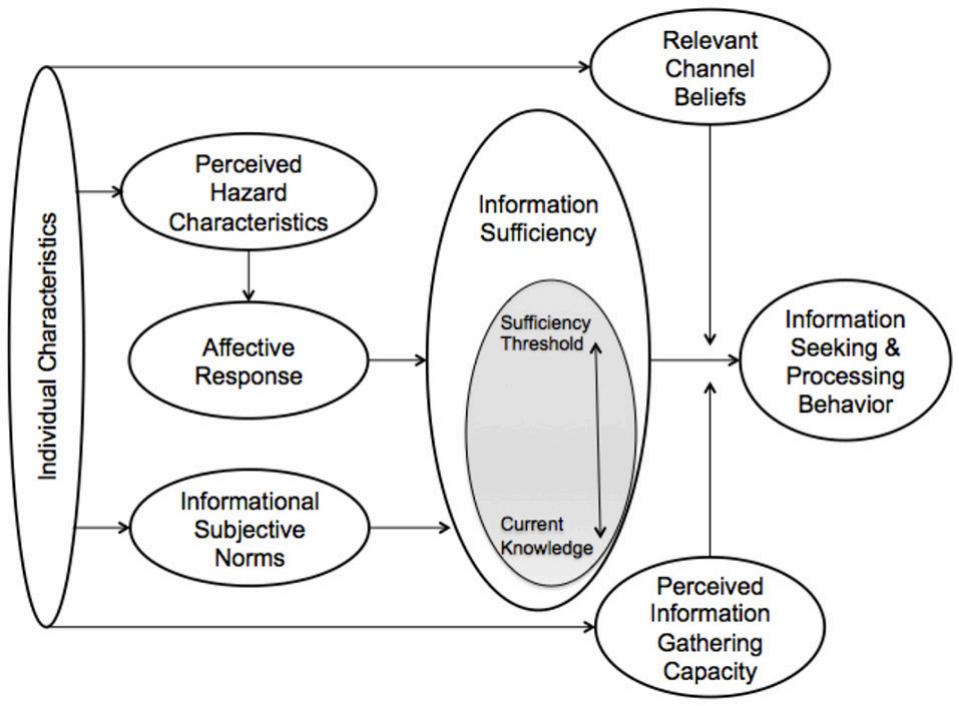
Risk Information Seeking and Processing Model [adapted from (6)].

**Figure 2:**
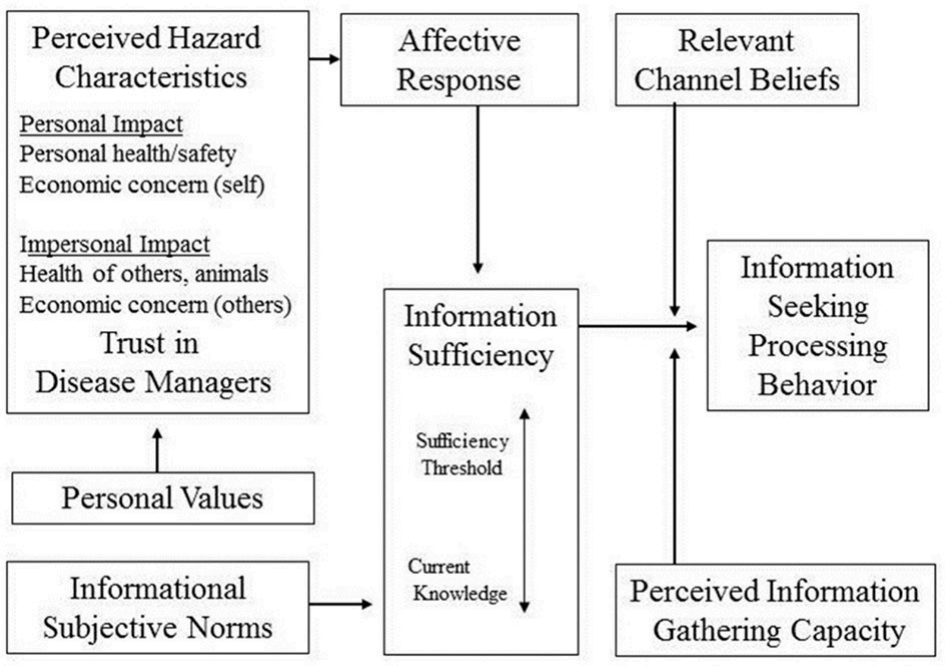
Clarke's RISP framework for zoonotic diseases [adapted from (1)].

According to the RISP model (Figure 1), an individual's perception of risk is driven by the degree to which they think they are informed about a threat and how he or she seeks out and processes information about the risk (6). Like many other risk perception models, Griffin's RISP model has been used to study health and personal risks. However, environmental risks have been a special emphasis of the RISP model research (18, 20, 21). Clarke (1) presented a modified RISP framework, which integrated values, to examine how individuals perceive zoonotic disease (such as bTB) as a threat to wildlife (Figure 2).

The central component that drives the RISP model is “information sufficiency” (6). In the process of developing the perception of a threat, an individual will assess how much information they currently have and evaluate that level based on how much information they think is necessary to understand the threat. If the individual believes they have insufficient information, they will seek out and process additional information about the topic. Demographics, such as age, income, education level, and personal importance of hunting, and informational subjective norms (the social pressure to be informed) may influence individuals' information sufficiency thresholds.

Affective responses may also have an important influence on information sufficiency, and the common affective responses studied by RISP frameworks are worry and anxiety (17). However, fear and anger are also possible responses that could apply, with the degree of perceived personal control influencing with one expresses fear (low control) or anger (high control) to a situation (6, 20). Subjective norms, an individual's assessment of whether his or her peers expect him or her to be informed, can also lead to the information sufficiency stage regarding the threat (6). Even if an individual is not concerned about a risk, they may decide to learn more if they think it will give them more information to talk about with peers.

Another set of components, “perceived information gathering capacity,” refers to whether the individual can understand (or comprehend) and access available information. Information that is too complicated or technical may discourage an individual from seeking more information about the risk. Relevant channel beliefs, which we did not collect data on, refer to the “channels,” or sources of information, through which an individual learns about a risk (6). In the model, relevant channel beliefs do not interact with other predictor variables and subsequent work on RISP excludes relevant channel beliefs (22). The information sources or amount of information an individual has access to may affect desire to seek information about the source.

These variables are meant to measure an individual's heuristic and systematic processing of information, and in the model, are hypothesized to influence information seeking behavior through the information sufficiency and perceived information gathering capacity variables, which share a direct relationship with information seeking (1, 6, 23). Systematic processing refers to higher order processing, which requires effort on the part of the individual and, more likely than heuristic processing, may lead to attitudinal change (24, 25). Heuristic processing occurs at a comparatively shallow level and uses superficial cues for interpreting information (25). The two forms of information processing are a major field of social-psychological research (26–28). In the context of understanding perceptions about bTB, the distinction is important as hunters who engage in heuristic processing may be more easily discouraged from hunting due to concerns about bTB than those who engage in systematic processing.

In addition to Griffin et al. (6) RISP model, we adopted components of Clarke's (1) Zoonotic Disease Risk Information Seeking and Processing (ZDRISP) framework. Following Clarke (1), we measured hunters' perceptions of the impact of bTB to themselves, other people, and wildlife. The framework includes components that examine how personal impact (e.g., health and financial costs to the individual) and impersonal impact (e.g., health and financial costs to other people, wildlife species, and society) can be included in a traditional RISP model. Clarke (1) also emphasizes the importance of trust in the managing agency on information seeking and processing. Low trust of an agency might discourage, or frustrate people, from learning about the threat. Trust will also likely have an important role in whether an individual supports the agencies policies to manage the threat. Kahlor (19), building off similar communication processing frameworks, argued for a more integrated RISP model that was termed, “A Planned Risk Information Seeking Model” (PRISM). The key aspect of PRISM is the integration of the core RISP model with conceptual components from the Theory of Planned Behavior (24, 29). These components include: (1) positive/negative evaluations of a behavior (attitudes); (2) perceptions of social pressure to engage in a behavior (subjective norms); and (3) perceived ability to engage in a behavior (perceived behavioral control) (19).

Given the controversial nature of wildlife disease management, there is a need to understand how stakeholders perceive, gain, and distribute information. The RISP model hypothesizes that information sufficiency and perceived information gathering capacity directly influence information seeking behavior. Because of the direct relationship of these variables to information seeking in the model (Figure 1), we expect them to demonstrate the greatest influence on information seeking behavior relative to other variables. Previous studies conducted using the RISP framework and its variants (1, 20, 19) guided the development of the survey instrument used in this research. We also collaborated closely with the Minnesota DNR regarding their bTB strategy related to deer management. The adoption of this RISP model for evaluating perceived threats from wildlife disease aligns with bTB disease research in Michigan (30), and we were particularly interested in potential insights from the application of the model in the two similar contexts.

## Methods

### Sampling

In Minnesota, deer are managed by permit area (*n* = 128) and hunters are asked to identify the areas they intend to hunt that year. Although hunters are not required to hunt only in the area they identify, previous research revealed that most return to the same location annually (31). We drew a proportional random sample from Minnesota's Electronic Licensing System license database of adult individuals who purchased a deer license and indicated they hunted in areas affected by bTB. Our survey followed a modified version of the Tailored Design Method (32). We mailed survey to 2,100 licensed hunters from the bTB affected permit areas (*n* = 7) and used three waves of mailing to maximize response rate. We collected data during late summer and fall of 2012 (University of Minnesota Institutional Review Board Study Number: 0609E92806).

### Measurement variables

#### Dependent variable

Following the RISP model, we wanted to understand what variables influenced the likelihood of individuals to actively seek out bTB information. We used five items developed and tested in previous studies of the RISP framework to measure information seeking (Table 1). We used a five-point scale ranging from 1 = “*strongly disagree*” to 5 = “*strongly agree*” for each item. Questions that were phrased to report avoidance of information seeking were reverse coded for reliability and subsequent scale formation. We computed the latent dependent variable, “information seeking behavior” as the mean score of the 5 scale items.

**Table 1:** Measurement of variables.

**Item**	**Mean**	**SD**	**alpha**
(A) Dependent - Information seeking behavior^1^	0.77
a) When the topic came up, I was likely to tune it out. (reverse coded)	3.79	0.89	
b) I'd go out of my way to avoid learning more about bovine TB management. (reverse coded)	4.08	0.84	
c) Gathering a lot of information about bovine TB management was a waste of time. (reverse coded)	3.83	0.93	
d) I tried to learn more about TB.	3.48	0.87	
e) I was likely to go out of my way to get more information about bovine TB management.	3.01	0.93	
(B) Independent	
1) Affective response^2^			NA
a) Anger	4.23	3.41	
b) Worry	4.65	3.36	
c) Fear	5.19	3.2	
2) Perceived impacts of bTB^3^			NA
a) Economic impacts to cattle producers	3.07	1.22	
b) Threats to the health of deer	3.81	1.03	
c) Reducing the deer population in the area	3.84	1.15	
d) Economic impacts to businesses that depend on deer hunting	3.23	1.16	
e) Threats to the health of other deer hunters from infected deer	2.74	1.32	
f) Reducing your deer hunting opportunity	3.72	1.2	
g) Threats to your personal health or family members from infected deer	2.68	1.4	
h) Financial costs to you personally	2.08	1.28	
3) Personal impact concerns^3^			0.71
a) Reducing the deer population in the area	0.48	0.65	
b) Reducing your deer hunting opportunity	0.58	0.59	
c) Threats to your personal health or family members from infected deer	0.45	0.68	
d) Financial costs to you personally	0.48	0.66	
4) Subjective norms^1^			0.74
a) People who are important to me thought I should stay on top of information about bovine TB	3.07	0.96	
b) People close to me expected me to get information about bovine TB	2.8	0.99	
c) Most of the people I know wanted to talk about bovine TB	2.99	1.18	
5) Perceived information gathering capacity^1^			0.74
a) I knew what questions to ask of the experts	2.77	0.96	
b) I knew where to go for information	3.39	0.95	
c) I could take the time to gather any information I needed	3.32	0.92	
d) Much of the information was too technical for me to understand (reverse coded)	3.44	0.87	
e) I could separate fact from fiction	3.86	0.86	
f) I could understand the information if I made the effort	2.77	0.7	
6) Attitude toward seeking information^4^			0.90
a) Worthless … valuable	5.18	1.35	
b) Foolish … wise	5.36	1.29	
c) Unhelpful … helpful	5.16	1.25	
7) Trust in DNR^1^			0.93
a) I trust the Minnesota DNR to manage bovine TB	3.14	1.14	
b) DNR officials are concerned about minimizing the impacts of bovine TB on deer hunters	3.43	1.09	
c) The Minnesota DNR does a competent job of minimizing the impacts of bovine TB	3.22	1.06	
d) The DNR is open and honest in the things they do in say when managing bovine TB	2.92	1.12	
e) The DNR makes decisions about managing bovine TB in a way that is fair	2.87	1.08	
f) The DNR listens to deer hunters' concerns when managing bovine TB	2.76	1.13	

1 Response options ranged from “1 - Strongly disagree” to “5 - Strongly agree.”

2 Scale ranged from 0 to 10, where “0 - None of this feeling” to “10 - A lot of this feeling.”

3 Response options ranged from “1 - Not at all concerned” to “5 - Extremely concerned.”

4 Response options ranged from “1 - Extremely worthless/foolish/unhelpful” to “7 - Extremely valuable/wise/helpful.”

#### Independent variables

In addition to respondent demographics, we constructed seven latent independent variables as identified in the RISP (6) and ZDRISP (1) models. We measured (1) demographic variables such as age, education level, and importance of hunting, and following Clarke (1), (2) we assessed respondents' general perceptions of bTB impact concerns, or risk judgment (8 items) and personal impact concerns (4 items). Each item was measured on a five-point scale ranging from 1 = “*not at all”* to 5 = “*extremely” concerned*. We also measured the (3) individual affective responses (anger, worry, fear) of study participants to the discovery of bTB and the DNR's subsequent management of the disease on an 11-point scale ranging from 0 = “*none of this feeling*” to 10 = “*a lot of this feeling*.” We measured (4) subjective norms using three items on a five-point scale from 1 = “*strongly disagree*” to 5 = “*strongly agree*,” and following Fishbein and Ajzen (33, 29), we used semantic differential scales to assess respondents' evaluation of seeking information about bTB management. Respondents were asked to evaluate (on a 7-point scale) whether their information seeking was worthless or valuable, foolish or wise, and unhelpful or helpful. We used six items to (5) define respondents' beliefs about their personal ability to obtain and understand bTB-related information. Responses related to information seeking capacity were also measured on a five-point style scale ranging from 1 = “*strongly disagree*” to 5 = “*strongly agree*.”

We also asked survey recipients about (6) their current knowledge and the amount of effort they dedicated to learning about bTB. We asked survey recipients to rate each on a scale ranging from 0 (no information) to 100 (all available information). Following Griffin et al. (20), we did not equate knowledge insufficiency as difference scores between these two measures but rather (7) regressed sufficiency threshold scores on initial knowledge scores to identify “information insufficiency” [as delineated in Cohen et al. (34)].

### Data analysis

Data were analyzed using the IBM Statistical Program for the Social Sciences (SPSS v. 20, 2013). (Any use of trade, firm, or product names is for descriptive purposes only and does not imply endorsement by the U.S. Government.) We grouped variables into their multi-item scales and tested internal consistency using Cronbach's alpha. Then we used hierarchical multiple regression models to examine the relationship between explanatory variables and the predictor, information seeking behavior as well as the ability of the RISP model to predict information seeking behaviors. We generated 7 separate models to test whether including each additional variable in our modified RISP framework improved the predictive ability of the model.

## Results

### Response rates and respondent characteristics

Of the 2,100 surveys mailed, 134 were undeliverable. For the remaining 1,966 surveys, 745 were completed and returned, resulting in a response rate of 38%. There was a wide distribution in the responses to survey items for variables included in the models, which suggested that our findings are likely to be representative of the sample population of hunters in all 7 deer permit areas impacted by bTB management. Most of survey respondents were male (93%) and half had completed some education above the high school level (Table 2). Many respondents did not respond to the income question (*n* = 211), which is typically a component of personal characteristics in the RISP model. Income, however, did not appear to influence final model results and excluding it from the model provided a larger sample size for analysis. In a simple regression with income predicting information seeking behavior, the two variables were not strongly correlated, with *r*_(519)_ = 0.008, *p* < 0.04. For this reason, we did not use income as an independent variable in the analysis. The final usable response rate for modeling was 30% (*n* = 598) after removing individuals excluded due to incomplete responses to survey items other than income.

**Table 2:** Respondent characteristics.

	**N**	**%**
**GENDER**		
Male	675	93.0
Female	51	7.0
**EDUCATION**		
Grade school	7	1.0
Some high school	10	1.4
High school diploma or GED	151	20.9
Some vocational or technical school	65	9.0
Vocational or technical school (associate's)	126	17.5
Some college	118	16.4
Four-year college	164	22.7
Some graduate school	29	4.0

### Scale reliability and model results

Within the RISP framework, our reliability analyses supported the creation of the latent dependent variable (alpha = 0.77) and the independent latent variables (Table 1). Multiple regression demonstrated significant effects of attitudes, subjective norms, information seeking capacity and information insufficiency on the information seeking behaviors of northwest Minnesota deer hunters (Tables 3, 4). The final model in the hierarchical regression suggested the RISP framework explained 43% of the variability in northwest hunters' information seeking behaviors in response to bTB occurrence (Table 3).

**Table 3:** RISP framework hierarchical regression models of self-reported information seeking behaviors in northwest Minnesota hunters, data from 2012.

**Predictor**	**SE**	**Std. β**	***t***	***p***	***R*^2^**	**Δ*R*^2^**	**F**	***p***
Model 1					0.050		10.4	< 0.001
Age	0.00	0.05	1.148	0.25				
Education	0.01	0.16	3.993	< 0.001				
Hunting importance	0.03	0.16	4.012	< 0.001				
Model 2					0.102		9.60	< 0.001
						0.053	8.64	< 0.001
Age	0.00	0.04	0.931	0.35				
Education	0.01	0.19	4.71	< 0.001				
Hunting importance	0.03	0.13	3.18	0.00				
Personal Impact	0.03	0.15	3.57	< 0.001				
Risk judgment	0.00	0.12	2.94	0.003				
Trust in DNR	0.03	0.09	2.20	0.029				
Self-efficacy	0.03	0.07	1.83	0.068				
Model 3					0.171		12.1	< 0.001
						0.068	16.1	< 0.001
Age	0.002	0.04	1.06	0.29				
Education	0.013	0.19	4.87	< 0.001				
Hunting importance	0.031	0.10	2.40	0.02				
Personal impact	0.031	0.10	0.211	0.83				
Risk judgment	0.000	0.08	1.97	0.05				
Trust in DNR	0.027	0.10	2.58	0.01				
Self-efficacy	0.025	0.08	1.99	0.47				
Anger	0.009	0.06	1.16	0.25				
Worry	0.014	0.30	4.11	< 0.001				
Fear	0.014	−0.04	−0.531	0.60				
Model 4					0.370		28.6	< 0.001
						0.199	92.4	< 0.001
Age	0.00	−0.02	−0.47	0.64				
Education	0.01	0.14	4.10	< 0.001				
Hunting importance	0.03	0.07	2.02	0.04				
Personal impact	0.03	−0.06	−1.58	0.12				
Risk judgment	0.00	0.03	0.79	0.43				
Trust in DNR	0.02	0.03	0.69	0.49				
Self-efficacy	0.02	0.02	0.65	0.52				
Anger	0.01	0.01	0.17	0.87				
Worry	0.01	0.15	2.22	0.03				
Fear	0.01	−0.02	−0.25	0.81				
Subjective norm	0.03	0.26	6.71	< 0.001				
Attitude	0.02	0.34	8.51	< 0.001				
Model 5					0.420		32.5	< 0.001
						0.050	50.7	< 0.001
Age	0.00	0.01	0.322	0.75				
Education	0.01	0.09	2.71	0.01				
Hunting importance	0.03	0.06	1.79	0.07				
Personal impact	0.03	−0.04	-952	0.34				
Risk judgment	0.00	0.02	0.507	0.61				
Trust in DNR	0.02	0.02	0.557	0.58				
Self-efficacy	0.02	−0.01	−0.411	0.68				
Anger	0.01	−0.01	−0.323	0.75				
Worry	0.01	0.16	2.57	0.01				
Fear	0.01	0.01	0.092	0.93				
Subjective norm	0.03	0.20	5.32	< 0.001				
Attitude	0.02	0.29	7.34	< 0.001				
Capacity	0.04	0.25	7.12	< 0.001				
Model 6					0.421		30.3	< 0.001
						0.001	0.848	0.357
Age	0.00	0.01	0.24	0.81				
Education	0.01	0.09	2.70	0.01				
Hunting importance	0.03	0.06	1.76	0.08				
Personal impact	0.03	−0.04	−1.00	0.32				
Risk judgment	0.00	0.01	0.43	0.67				
Trust in DNR	0.02	0.02	0.67	0.50				
Self-efficacy	0.02	−0.01	−0.43	0.67				
Anger	0.01	−0.02	−0.43	0.67				
Worry	0.01	0.16	2.56	0.01				
Fear	0.01	0.01	0.16	0.87				
Attitude	0.03	0.20	5.10	0.00				
Subjective norm	0.02	0.29	7.32	0.00				
Capacity	0.04	0.25	6.83	0.00				
Current knowledge	0.00	0.03	0.921	0.35				
Model 7					0.427		28.9	< 0.001
						0.006	6.02	0.014
Age	0.00	0.01	0.43	0.67				
Education	0.01	0.09	2.54	0.01				
Hunting importance	0.03	0.06	1.79	0.07				
Personal impact	0.03	−0.04	−0.97	0.33				
Risk judgment	0.00	0.01	0.25	0.80				
Trust in DNR	0.02	0.03	0.83	0.41				
Self-efficacy	0.02	−0.01	−0.20	0.84				
Anger	0.01	−0.02	−1.55	0.58				
Worry	0.01	0.16	2.50	0.01				
Fear	0.01	0.01	0.12	0.90				
Attitude	0.03	0.20	5.18	0.00				
Subjective norm	0.02	0.26	6.28	0.00				
Capacity	0.04	0.25	6.83	0.00				
Current knowledge	0.00	0.03	0.74	0.46				
Information insufficiency	0.00	0.09	2.45	0.01				

**Table 4:** Sources of information.

	***N***	**Mean**	**SD**
Radio news	718	2.43	0.955
Television news	712	2.33	0.943
Local newspapers	717	2.83	0.981
Statewide newspapers and news magazines	714	2.56	1.005
Internet sources	705	2.30	1.137
Minnesota DNR	716	2.74	1.005
Minnesota board of animal health	704	1.74	0.997
Family, friends, social network	719	2.86	0.888
Public meetings	707	1.65	0.951

Respondents were asked “From what sources did you get information about TB and bovine TB management?”

*Responses were “1” (Not at all), “2” (Slightly), “3” (Moderately), “4” (Very Much)*.

Model 1 included only individual characteristics and explained a small amount of the variability in information seeking behaviors (6.5%) (Table 3). The addition of variables pertaining to personal impact, risk judgment, trust in DNR, and self-efficacy (Model 2) resulted in a ΔR^2^ of 5.3% (Table 3). Affective response (Model 3) slightly increased the amount of explained variability in information seeking behaviors (ΔR^2^ = 6.8%; R^2^ = 17.1%). Subjective norms and attitudes (Model 4) more than doubled the amount of explained variability in the RISP model (ΔR^2^ = 19.9%; R^2^ = 37.0%). Current knowledge (Model 5) increased R^2^ by 5.0%; whereas the addition of information insufficiency (Model 6) changed the amount of explained variability minimally (ΔR^2^ = 0.1%). Each iteration of our global model was statistically significant.

In model 7, findings suggest only education, worry, subjective norm, information seeking capacity, and information insufficiency are statistically significant (*p* < 0.05) predictors of information seeking behaviors in the RISP model (Table 3). Information seeking capacity (*p* < 0.001) and information insufficiency (*p* = 0.019) were significant predictors of information seeking behaviors of northwest Minnesota deer hunters, after controlling for individual characteristics, perceived hazard characteristics, affective response, attitudes and informational subjective norms as the model suggests. As the variables most proximal to information seeking behavior in the RISP framework, the result is expected (Figure 1). However, while information insufficiency was significant (*p* < 0.05), the amount of additional variability it explains in the model was small (< 1%). Changes in the amount of variability explained in the models are greatest with the addition of subjective norms and attitudes (19.9%) and affective response (6.8%) (Table 3).

In addition to the application of the RISP framework, we found that northwest Minnesota hunters reported their family, friends, and social network as their greatest source of information about bTB and bTB management, followed by statewide newspapers and news magazines. Respondents considered state agencies and public meetings as the least-utilized information source, except for the Minnesota Department of Natural Resources which had the third highest average response (Table 4).

## Discussion

Our study aimed to better understand the information seeking behaviors of deer hunters regarding disease management. Specifically, we use the RISP model to investigate the relationships of individual variables on information seeking and processing behavior. To achieve this, we used hierarchical multiple regression analysis utilizing the RISP framework. Results revealed several discrepancies from the expectations of the RISP model and from studies using the RISP model in other contexts. These include research on topics ranging from environmental risks to the health communication sciences (20, 19, 30).

Unlike the findings of Kahlor's PRISM research (19), our application of the RISP model variable “information sufficiency” was statistically significant. However, its importance in predicting information seeking is diminished by its lack of predictive power. The addition of information sufficiency in the RISP model yielded almost no increase in variance in information seeking behavior explained (< 1%). Our finding differs from our original expectation—that variables most proximal to information seeking behaviors in the model would explain the greatest proportion of variation.

In our model of information seeking behaviors, we found that attitudes explained the greatest amount of variability in information seeking behaviors of northwest Minnesota hunters. We believe this relates to the time that elapsed between the survey implementation and the initial outbreak of bTB in MN. Bovine TB was originally detected in a wild deer in 2005, and this research was conducted in the summer of 2012. During that time, the Minnesota DNR implemented aggressive deer control efforts that resulted in a 60% decrease in the population, which likely increased negative attitudes toward bTB management and the agency in general.

Our findings pertaining to attitude and social norms exerting a strong influence on risk behaviors of individuals are similar to other applications of the model in the context of zoonotic disease risk perception [e.g., 30)].

Because the RISP model has been widely applied in other contexts and may be useful to natural resource managers in the future, it is important to understand its operation in an applied setting. This research suggests that using the complete RISP model to explain behaviors after the immediate onset of a threat, or once a threat has been eradicated, may be challenging. Information about the extent of disease was readily available by the time we surveyed hunters, and they were probably better informed about past risks (and the lack of present risk) from bTB than at the time of the initial disease detection. Model variables that are stable across time (e.g., demographics and attitudes) appear to be primary drivers (e.g., explain the greatest amount of variability). In particular, we believe that using the Theory of Planned Behavior (24) might provide a more parsimonious model in cases where the disease has been present for a substantial time, communication has been present, and attitudes, norms, and beliefs are likely the drivers of whether or not people will seek out and use information. In a case where the disease threat is relatively new and most people are unlikely to have had a lot of exposure to information about the threat, information insufficiency might be more of a driver soon after disease outbreak.

## Conclusions, limitations, and implications

Due to early detection of the disease and aggressive management actions, the prevalence of bTB in Minnesota never reached levels observed in Michigan, where bTB eradication from the wild population of white-tailed deer is unlikely (35). As such, Minnesota provides a case study for successful bTB management (if “success” is measured as no longer detecting positive animals). Although Minnesota received classification as a bTB-free state in 2011, the possibility of future occurrence of bTB or other wildlife disease outbreaks remains. Understanding how hunters perceive bTB and bTB management, as well as what motivates them to attend to information concerning the risks and management of bTB, is integral in creating socially acceptable policy to manage for future occurrences of bTB or similar zoonotic diseases affecting humans and wildlife (3, 35).

This project explores the use of the RISP model in the context of wildlife disease and management. The findings about the operation of the RISP theory in an applied context inform future research and management, indicating that in this instance attitudes and norms exert greater influence on hunters' information seeking behaviors than the RISP framework appears to suggest. Evidence suggests that successful natural resource management and policy implementation requires stakeholder support, especially from hunters and private landowners (12). Communicating zoonotic disease risks to the public, as in the case of bTB, proves challenging for managers (12). The findings from this study might appear to have little practical utility to wildlife management agencies that primarily focus on “scientific management” largely based in the biological sciences. The variables influencing risk perceptions and information seeking behaviors (attitudes and social norms) seem out of the control of managers. As noted by Riley et al. (36), however, effective wildlife management in the twenty-first century might require complex integration of biological and human dimensions information. Such integration is likely to move wildlife disease management decisions and actions away from only attempting to address the biological issues that appear to be under the control of “scientific management” toward also understanding and addressing the psychological and social phenomena associated with the stakeholders for whom agencies are managing the wildlife resource.

When this research is contextualized in this more complex management setting, its benefits to managers are more apparent. Clearly understanding the prevalent attitudes and subjective norms of stakeholder and communities impacted by bTB can assist in developing messages for communicating disease risks as well as management actions. In previous natural research management studies using the Theory of Reasoned Action or Theory of Planned Behavior, careful analysis of the beliefs influencing attitudes and norms have assisted with understanding stakeholders and developing messages and communication strategies. These studies have been conducted in diverse contexts including reintroduction of wolves (37), habitat conservation for endangered species (38), the lethal control of deer populations for conservation (13, 14) and support for limiting use of lead shot (39, 40). Our study demonstrates that the use of similar models holds promise for better understanding what influences stakeholder behaviors related to wildlife disease management. Decisions that clearly take into consideration the impacts to stakeholders may enhance the social acceptance of risk management actions and processing of communication, ultimately bettering relationships with stakeholders and improving policy outcomes.

We also noted several limitations of our study, mostly related to the time between initial detection (2005) and survey implementation (2012). Asking respondents to rate their affective responses when they initially heard about the outbreak limits our findings; there may have been inaccuracies in participants' recollections of their emotions upon hearing of bTB. We also expect that survey participants used hindsight to inform their responses related to perceptions of threats from bTB. We also asked people to recall in the past how they perceived threats of bTB after it had already been eradicated from the state. Had we surveyed hunters immediately following the onset of bTB, rather than after Minnesota was declared bTB free, respondents' likelihood of reporting perception of a threat from bTB may have been higher. Future research should attempt to minimize the duration between event and research. Additionally, while our response rate was not unusual for such surveys, the fact that most hunters (62%) we contacted did not respond to the survey may indicate a lack of concern, or apathy, about the topic.

## Author contributions

DF and LC designed research. DF, LC, and AH performed research and collected data. MC, DF, LC analyzed data. All authors wrote and reviewed the paper.

### Conflict of interest statement

The authors declare that the research was conducted in the absence of any commercial or financial relationships that could be construed as a potential conflict of interest. The reviewer AZ declared a past co-authorship with one of the authors AH to the handling Editor. The reviewer AZ declared a shared affiliation, with no collaboration, with one of the authors, MC, to the handling Editor.
